# Comparative Study of the Temperature Sensitive, Cold Adapted and Attenuated Mutations Present in the Master Donor Viruses of the Two Commercial Human Live Attenuated Influenza Vaccines

**DOI:** 10.3390/v11100928

**Published:** 2019-10-10

**Authors:** Laura Rodriguez, Pilar Blanco-Lobo, Emma C. Reilly, Tatsuya Maehigashi, Aitor Nogales, Andrew Smith, David J. Topham, Stephen Dewhurst, Baek Kim, Luis Martínez-Sobrido

**Affiliations:** 1 Department of Microbiology and Immunology, University of Rochester, Rochester, New York, NY 14642, USA; laurita85oviedo@hotmail.com (L.R.); piblanlo@gmail.com (P.B.-L.); emma_reilly@urmc.rochester.edu (E.C.R.); nogales.aitor@inia.es (A.N.); Andrew_Smith72@URMC.Rochester.edu (A.S.); David_topham@urmc.rochester.edu (D.J.T.); stephen_dewhurst@me.com (S.D.); 2 David H. Smith Center for Vaccine Biology and Immunology, University of Rochester, Rochester, New York, NY 14642, USA; 3 Center for Drug Discovery, Department of Pediatrics, School of Medicine, Emory University, Atlanta, Georgia, GA 30322, USA; t.maehigashi@emory.edu (T.M.); baek.kim@emory.edu (B.K.); 4 Medical Scientist Training Program (MSTP), University of Rochester, Rochester, New York, NY 14642, USA; 5 College of Pharmacy, Kyung-Hee University, Seoul 02447, Korea

**Keywords:** influenza, vaccine, live-attenuated vaccine

## Abstract

Influenza viruses cause annual, seasonal infection across the globe. Vaccination represents the most effective strategy to prevent such infections and/or to reduce viral disease. Two major types of influenza vaccines are approved for human use: inactivated influenza vaccines (IIVs) and live attenuated influenza vaccines (LAIVs). Two Master Donor Virus (MDV) backbones have been used to create LAIVs against influenza A virus (IAV): the United States (US) A/Ann Arbor/6/60 (AA) and the Russian A/Leningrad/134/17/57 (Len) H2N2 viruses. The mutations responsible for the temperature sensitive (*ts*), cold-adapted (*ca*) and attenuated (*att*) phenotypes of the two MDVs have been previously identified and genetically mapped. However, a direct comparison of the contribution of these residues to viral attenuation, immunogenicity and protection efficacy has not been conducted. Here, we compared the In vitro and in vivo phenotype of recombinant influenza A/Puerto Rico/8/34 H1N1 (PR8) viruses containing the *ts, ca* and *att* mutations of the US (PR8/AA) and the Russian (PR8/Len) MDVs. Our results show that PR8/Len is more attenuated in vivo than PR8/AA, although both viruses induced similar levels of humoral and cellular responses, and protection against homologous and heterologous viral challenges. Our findings support the feasibility of using a different virus backbone as MDV for the development of improved LAIVs for the prevention of IAV infections.

## 1. Introduction

In the United States (US), the Centers for Disease Control and Prevention (CDC) has estimated that influenza has caused between 9.3 and 49.0 million illnesses each year, since 2010- along with between 12,000 and 79,000 deaths annually [1]. Two types of influenza cause seasonal, global epidemics in human populations each year: influenza A virus (IAV) and influenza B virus (IBV) [2,3]. IAVs can also cause sporadic pandemics of great consequences when new viruses are introduced into humans [4].

Vaccination is the most cost-effective approach to prevent influenza infections [5]. Currently, three major influenza vaccines are approved for human use in the US: recombinant (r) hemagglutinin (HA) protein, inactivated influenza vaccines (IIVs) and live attenuated influenza vaccines (LAIVs) [1]. IIVs are delivered by the intramuscular route and principally induce humoral responses against the viral HA protein [6,7,8], one of the major antigenic determinants of influenza viruses which is responsible for viral entry [9]. On the other hand, LAIVs are administrated intranasally (mimicking the natural route of infection) and induce both humoral and cellular immune responses in the mucosa [10,11,12,13]. In the US and the European Union, the age indication for both vaccines is different: in general, IIVs are used in children 6 months of age and in all adults; and LAIVs are used in individuals between 2 to 49 years old, with the exception of pregnant woman and/or immunocompromised people [1]. However, since the introduction of the quadrivalent vaccine formulation during the 2013–2014 influenza season, different studies revealed less than expected effectiveness of the LAIV and, therefore, a recommendation of the IIVs over LAIV over IIVs.

LAIVs have been generated by passaging influenza viruses at gradually reduced temperatures in eggs or cultured cells to generate temperature sensitive (*ts*) viruses that replicate efficiently at low temperatures (cold adapted, *ca*) but not at elevated temperatures (temperature sensitive; *ts*); such viruses also possess an attenuated phenotype (*att*) in vivo [14,15,16,17,18]. The generation of these viral variants was based on the ability of influenza viruses to replicate in the upper (25–33 °C) and lower (37 °C) respiratory tract (URT and LRT, respectively). LAIVs have restricted replication at non-permissive temperatures encountered in the LRT (37 °C–39 °C) but are able to replicate efficiently at permissive temperatures encountered in the URT and nose (33 °C). As a consequence, they induce mucosal immunity at the site of infection (URT) [11,12,13,19,20,21], as well as robust humoral [11,12,13,21,22,23,24] and cellular [22] immunity -leading to the recruitment of influenza-specific CD8 T cells into the lungs and protection against subsequent infections [11,12,13,25,26,27,28].

The traditional methods for generating seasonal LAIVs is based on the co-infection of embryonated chicken eggs with a Master Donor Virus (MDV) and the seasonal virus recommended by the CDC and international health agencies to be part of the vaccine [29]. The viral passages are carried out at low temperatures (25–26 °C) [29] and the reassortant virus (6 + 2) containing the six internal genes of the MDV (PB2, PB1, PA, NP, M and NS) and the HA and NA from the seasonal viral strain is cloned by limited dilution in the presence of antibodies against the surface glycoproteins (HA and NA) of the MDV strain. Thus, all LAIVs share the same internal genes (derived from the MDV) and differ only in their HA and NA components. More recently, reverse genetics techniques have been used to generate seasonal LAIVs (e.g., Flumist^®^ Quadrivalent Vaccine, AstraZeneca).

Two approaches have been used to develop seasonal IAV LAIVs that differ in the MDV: (1) the US A/Ann Arbor/6/60 H2N2 LAIV (AA) that is commercialized under the names of FluMist in the US and Canada, and Fluenz in the European Union; and (2) the Russian A/Leningrad/134/17/57 H2N2 LAIV (Len) that is commercialized under the names Ultravac (Russia) and Nasovac-S (India) [30] and also used in China and Thailand [31]. In the case of the US AA MDV, the mutations responsible for the *ts, ca* and *att* phenotype have been mapped to PB2 (N265S), PB1 (K391E, E581G and A661T) and NP (D34G) [32,33]. In the case of the Russian Len MDV, the mutations responsible for the *ts, ca* and *att* phenotype have been mapped to PB2 (V478L), PB1 (K265N and V591I) and NEP (M100I) [34]. Introduction of the US AA MDV mutations into the genomes of IAVs such as A/Puerto Rico/8/34 H1N1 (PR8) [35,36], A/canine/NY/dog23/09 H3N8 [11,12,21] or A/equine/Ohio/1/03H3N8 [13] resulted in similar *ts, ca* and *att* phenotypes as that of the original US AA MDV. However, introduction of the mutations of the US AA MDV into the pandemic A/California/04/09 H1N1 virus (Cal/09) resulted in reduced *ts* and *ca* in vitro and limited attenuation in vivo [37,38,39]. These results suggest that the *ts, ca* and *att* phenotypes induced by the mutations of the US AA MDV are influenced by the virus backbone into which they are introduced. Although some groups have generated recombinant viruses by using the internal genes of AA or the Len (with the LAIV mutations) as MDVs with different HA and NA combinations [40,41,42], a direct comparison of the contribution of the US and Russian MDVs residues to viral attenuation, immunogenicity and protection efficacy in the same viral genetic background has not been conducted.

Here, we compared the contribution of the mutations present in the US AA and the Russian Len MDVs using the backbone of influenza PR8 In vitro and in vivo. Our results show that PR8 containing the mutations of the Russian Len MDV (PR8/Len) is more attenuated in vivo than the PR8 containing the mutations of the US AA MVD (PR8/AA). However, both PR8/AA and PR8/Len induced similar levels of humoral and cellular responses and both induce protection against homologous (PR8, H1N1) and partially heterologous (X31, H3N2) viral challenges. Collectively, these findings support the feasibility of using the mutations of the US AA or the Russian Len MDVs in the PR8 virus backbone for the development of novel and improved LAIVs for the prevention of IAV infections. The use of circulating IAVs as MDVs in new LAIVs could grant a higher level of protection by the induction of more robust cellular immune responses against internal viral proteins (current H2N2 AA and Len LAIV backbones were isolated in 1960 and 1957, respectively). In addition, our findings have an important impact on the development and implementation of LAIVs with high levels of attenuation.

## 2. Materials and Methods

### 2.1. Cells and Viruses

Human embryonic kidney 293T (HEK293T; ATCC CRL-11268) and Madin-Darby canine kidney (MDCK; ATCC CCL-34) cells were maintained in Dulbecco’s modified Eagle’s medium (DMEM; Mediatech, Inc.) supplemented with 10% fetal bovine serum (FBS), and 1% PSG (penicillin, 100 units/mL; streptomycin 100 μg/mL; l-glutamine, 2 mM) at 37 °C with 5% CO_2_ as described [12].

The recombinant A/Puerto Rico/8/34 H1N1 (PR8) virus containing the *ts*, *ca* and *att* mutations of the Russian MDV A/Leningrad/134/17/57 LAIV (PR8/Len) was generated using previously described plasmid-based reverse techniques [43]. The recombinant PR8 wild-type (PR8/WT) and the mutant virus containing the *ts, ca*, and *att* mutations of the USA/Ann Arbor/6/60 H2N2 (PR8/AA) are described elsewhere [35,44]. All viruses were propagated in MDCK cells at 33 °C. A recombinant virus containing the HA and NA viral segments of influenza A/Hong Kong/1/1968 H3N2 in the background of PR8 virus (X31) was also used in our studies [45,46].

For viral infections, viral stocks were diluted in phosphate buffered saline (PBS) containing 0.3% bovine albumin (BA) and 1% penicillin and streptomycin (PS) (PBS/BA/PS). After 1 h viral adsorption at room temperature (RT), MDCK cells were maintained in post-infection (p.i.) DMEM media supplemented with 0.3% BA, 1% PSG, and 1 μg/mL of *N*-tosyl-l-phenylalanine chloromethyl ketone (TPCK)-treated trypsin (Sigma). Viral titers were determined by immunofocus assay (fluorescent forming units, FFU/mL) in MDCK cells at 33 °C using a monoclonal antibody (mAb) against influenza virus nucleoprotein, NP (ATCC HB-65, HL16-L10-4R5); as described [46].

### 2.2. Plasmids

To generate PR8/Len, the PR8/WT PB2, PB1 and NS viral segments were subcloned into a pUC19 plasmid (New England BioLabs) to introduce, using site-directed mutagenesis, the mutations responsible for the *ts, ca* and *att* phenotypes of the MDV A/Leningrad/134/17/57 H2N2 LAIV: PB2 V478L; PB1 K265N and V591I; and NEP M100I. Mutations were confirmed by sequencing (ACGT). Mutated PB2, PB1 and NS PR8/Len viral segments were subcloned from the shuttle pUC19 vector into the ambisense pDZ plasmid for virus rescue with the rest of the PR8 viral genes [35,47]. The PR8 PB2 and PB1 pDZ plasmids containing the *ts, ca* and *att* mutations of the MDV A/Ann Arbor/6/60 H2N2 LAIV: PB2 N265S; PB1 K391E, E581G and A661T were previously described [35].

### 2.3. Minigenome Assays

The ability of PR8/WT, PR8/AA and PR8/Len viral polymerase complexes to replicate and transcribe at different temperatures (33 °C, 37 °C and 39 °C) were evaluated in HEK293T cells (12-well plate format, 5 × 10^5^ cells/well, triplicates). Cells were co-transfected in suspension, using Lipofectamine 2000 (Invitrogen, Carlsbad, CA, US), with 250 ng of the indicated combinations of ambisense pDZ-PB2 (WT, AA or Len), pDZ-PB1 (WT, AA or Len), pDZ-PA and pDZ-NP plasmids, together with 500 ng of two reporter viral (v)RNA-like expression plasmids encoding the GFP and Gluc driven by a human RNA polymerase I promoter (hPol-I-GFP and hPol-I-Gluc, respectively), and 100 ng of a reporter plasmid expressing Cypridina luciferase (Cluc) under the control of the constitutively active simian virus 40 (SV40) promoter (pSV40-Cluc) to normalize transfection efficiencies. Cells transfected in the absence of pDZ-NP were included as a negative control. Empty pDZ plasmid was used to keep the amount of transfected plasmid DNA constant in the negative control. At 24 h post-transfection (p.t.), cells were analyzed for GFP expression and photographed using a fluorescent microscope (Olympus IX81) and camera (QIMAGING, Retiga 2000R); Gluc and Cluc expression levels were determined in tissue culture supernatants (TCS) using the BioluxGaussia and Cypridina Luciferase Assay kits (New England BioLabs) and quantified with a Lumicount luminometer (Packard). Reporter Gluc gene activation was normalized to that of Cluc and shown as fold induction over the level of induction of the negative control (no pDZ-NP). Mean values and standard deviations (SDs) were calculated and statistical analysis was performed using a two-tailed Student *t*-test with Microsoft Excel software. Data are represented as relative activity considering the activity of each polymerase complex combination at 33 °C as 100%.

### 2.4. Virus Rescue

PR8/Len virus rescue was performed as described for PR8/WT and PR8/AA viruses [11,12,43,46]. Briefly, co-cultures (1:1) of HEK293T and MDCK cells (6-well plate format, 1 × 10^6^ cells/well, triplicates) were co-transfected in suspension, using Lipofectamine 2000, with 1 μg of the eight-ambisense PR8 pDZ-PB2-Len, -PB1-Len, -PA, -HA, -NP, -NA, -M, and -NS-Len plasmids. At 12 h p.t., medium was replaced with p.i. medium and TCS were collected at three days p.t., clarified, and used to infect fresh monolayers of MDCK cells. After three days p.i., PR8-Len virus was plaque purified and scaled up using MDCK cells at 33 °C, as described [43].

### 2.5. Virus Growth Kinetics

Multicycle growth kinetics were carried out by infecting MDCK cells (12-well plate format, 5 × 10^5^ cells/well, triplicates) with PR8/WT, PR8/AA and PR8/Len at a multiplicity of infection (MOI) of 0.001. After 1 h of viral adsorption at RT, infection medium was replaced by p.i. medium and plates were incubated at different temperatures (33 °C, 37 °C and 39 °C). TCS were collected at the indicated times p.i. and viral titers were determined by immunofocus assay (FFU/mL) in MDCK cells as indicated before [46]. Mean values and SDs were calculated using Microsoft Excel software.

### 2.6. Plaque Assays

Monolayers of MDCK cells (6-well plate format, 1 × 10^6^ cells/well) were infected with PR8/WT, PR8/AA and PR8/Len for 1 h at RT, overlaid with agar, and incubated at 33 °C, 37 °C, or 39 °C [13]. At three days p.i., cells were fixed for 1 h at RT with 4% paraformaldehyde (PFA) and the overlays were removed. Cells were then permeabilized (0.5% Triton X-100 in PBS) for 15 min at RT and immunostained using the anti-NP mAb HB-65 and Vector kits (Vectastain ABC Vector kits and DAB HRP substrate kit; Vector) according to the manufacturer’s specifications.

### 2.7. Animal Experiments

Six-to-eight-week-old female C57BL/6 mice were purchased from the National Cancer Institute (NCI) and maintained in the animal care facility at the University of Rochester under specific pathogen-free conditions. All animal protocols were approved by the University Committee of Animal Resources (UCAR-2014-019E, approval date 08/29/2919) and complied with the recommendations in the Guide for the Care and Use of Laboratory Animals of the National Research Council [48]. To evaluate the in vivo attenuation of PR8/AA and PR8/Len viruses, mice (*N* = 5) were anesthetized intraperitoneally (i.p.) with 2,2,2-tribromoethanol (Avertin; 240 mg/kg of body weight) and inoculated intranasally (i.n.) with 30 μL of a virus preparation containing the indicated doses of virus diluted in PBS [49]. Mice were monitored daily for 14 days for morbidity (body weight loss) and mortality (survival). Mice showing 25% loss of their initial body weight were considered to have reached the experimental endpoint and were humanely euthanized [49]. The 50% mouse lethal dose (MLD_50_) was determined using the Reed and Muench method [50]. To evaluate virus replication, mice (*N* = 6) were anesthetized and infected as described and viral titers in homogenized lungs at 2 (*N* = 3) and 4 (*N* = 3) days p.i. were determined by immunofocus assay (FFU/mL) as previously described in MDCK cells at 33 °C [11,12,13,21,49]. The mean values and SDs were calculated using Microsoft Excel software.

For the vaccination and challenge experiments, 6–8-week-old female C57BL/6 mice (*N* = 5) were anesthetized and vaccinated i.n. with the indicated doses and viruses. A group of mice (*N* = 5) was also mock vaccinated with PBS as negative control. At fourteen days post-vaccination, mouse sera were collected by submandibular bleeding to evaluate the presence of total influenza antibodies (Abs) by enzyme-linked immunosorbent assay (ELISA) and hemagglutination inhibiting antibodies by hemagglutination inhibition (HAI) assay [49]. Twenty-four hours after bleeding, animals were challenged i.n. with the indicated dose of homologous (PR8) or heterologous (X31) WT viruses. After challenge, mice were monitored daily for 14 days for morbidity (body weight loss) and mortality (survival) as described above. Viral titers in the lungs of challenged mice were also calculated (see above). In the homologous (PR8) challenge, and to compare the differences in the protection efficacy provided by PR8/AA and PR8/Len viruses, we used a high dose (1000 MLD_50_) of WT PR8. For the heterologous challenge, since the majority of the neutralizing antibodies induced after LAIV vaccination are directed against the viral surface HA and NA glycoproteins that are different between our vaccine PR8 viruses and the challenge X31 virus, a low viral dose (50 MLD_50_) was used in these experiments.

### 2.8. Enzyme-Linked Immunosorbent Assays (ELISAs)

We evaluated the levels of total virus-specific Abs in the sera of vaccinated mice by ELISA as described [11,21,38,46,49,51]. Briefly, 96-well plates were coated with cell lysates from mock-, PR8/WT- and X31-infected MDCK cells or with 250 ng/well of recombinant PR8 HA protein (NR-19240; BEI Resources) and incubated ON at 4 °C. Mouse sera were tested as two-fold dilutions (starting dilution of 1:50) and titers determined as described [46,49].

### 2.9. Hemagglutination Inhibition (HAI) Assays

To evaluate the presence of hemagglutination inhibiting antibodies, mouse sera were treated with receptor-destroying enzyme (RDE; Denka Seiken) for 16 h at 37 °C and heat inactivated for 30 min at 56 °C. Then, sera were serially 2-fold diluted (starting dilution of 1:50) in 96-well V-bottom plates and mixed 1:1 with 4 hemagglutinating units (HAU) of the indicated viruses during 30 min at RT. HAI titers were determined by adding 0.5% turkey red blood cells to the virus-Ab mixtures followed by a 30 min incubation on ice [11]. The geometric mean titers and SDs from individual mice (*N* = 5) were calculated from the last well where hemagglutination was inhibited [49].

### 2.10. Evaluation of T Cell Responses

Six-to-eight-week-old female C57BL/6 mice (N = 5) were anesthetized and immunized with 10^3^ FFU of PR8/AA or with 10^3^ and 10^4^ FFU of PR8/Len, or mock- vaccinated with PBS. At 10 days p.i., lungs and spleens were collected and processed to obtain cellular preparations. Lungs were perfused with PBS before being surgically removed and dissociated in Gentle MACS (MiltenyiBiotek) C tubes using the Lung01 program. Samples were incubated in 5 mL (2 μg/mL) of Collagenase II in RPMI-1640 medium containing 8% FBS for 30 min at 37 °C with gentle agitation every 10 min. After digestion, samples were further dissociated using the Heart01 program. Cell suspensions were filtered through 70 μm filters prior to 75:40 Percoll (GE Healthcare) discontinuous gradient separation. The top layer (containing fat and other debris) was eliminated by aspiration. The cell layer was then harvested and washed, prior to counting and staining. Single-cell suspensions were prepared from collected spleens by disruption in RPMI +8% FBS; yields were quantitated by viable cell counts using Trypan blue exclusion on a hemocytometer.

Single cell suspensions were prepared for Flow cytometry analysis by staining in PBS containing 1% FBS, purified CD16/32 (clone 2.4G2), NP and PA tetramers, and the following antibodies: TCRβ-PerCPCy5.5, CD8α-FITC, CD4-BV421, CD44-APCCy7, and CD62L-PECy7. Cells were subsequently stained for viability using Live Dead Aqua (Invitrogen). All antibodies were obtained from eBioscience, BD Biosciences, or Biolegend. PA and NP tetramers [11,12,25,52] were obtained from the NIH tetramer core facility (Atlanta, GA). Cells were analyzed by an LSRII (BD Biosciences) in the University of Rochester Flow Cytometry core facility and analyzed using FlowJo software (Tree Star).

## 3. Results

### 3.1. Mutations of the US AA MDV and Russian Len MDV Confer Different Levels of Temperature Sensitivity to the Polymerase of PR8

**Figure 1:**
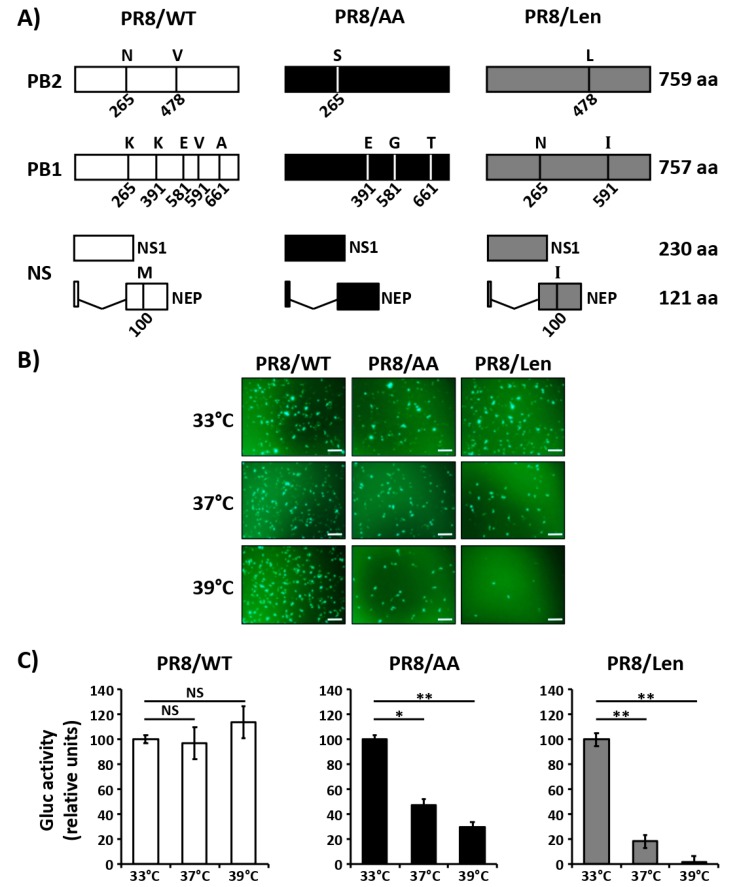
Effect of temperature on the polymerase activity of PR8/AA and PR8/Len. (**A**) Schematic representation of segments 1 (PB2), 2 (PB1) and 8 (NS) of PR8/WT (white, left), PR8/AA (black, middle) and PR8/Len (grey, right). Amino acid substitutions to generate PR8/AA and PR8/Len are indicated. B-C) Minigenome activity: HEK293T cells (12-well plate format, 4 × 10^5^ cells/well, triplicates) were transiently co-transfected with 250 ng of ambisense pDZ expression plasmids encoding the minimal requirements for viral genome replication and gene transcription (PB2, PB1, PA, and NP) together with 500 ng of a vRNA-like expression plasmid encoding GFP (**B**) or Gluc (**C**) under the control of the human polymerase I promoter (hPol-I-GFP and -Gluc, respectively), and 100 ng of an SV40 Cluc expressing plasmid to normalize transfection efficiencies. After transfection, cells were placed at 33 °C, 37 °C, or 39 °C, and viral replication and transcription was evaluated 24 h later by GFP (**B**) and luciferase (**C**) expression. Gluc activity was normalized to that of Cluc. Data represent means and SDs of the triplicates. Normalized reporter expression is relative to that obtained in the absence of pDZ NP. Data were represented as relative activity considering the activity of each polymerase complex at 33 °C as 100%. *, *P* < 0.05; **, *P* < 0.01; NS, no statistical differences (Student *t* test). Scale bars, 200 μm.

In a previous work [35], we introduced four of the five mutations (NP already contains a G at position 34) responsible for the *ts, ca* and *att* phenotypes of the US AA MDV [32,33] into the PB2 (N265S) and PB1 (K391E, E581G and A661T) genes of PR8 to create a PR8 LAIV (named henceforth PR8/AA) (Figure 1A). In vitro, these mutations affected viral replication at non-permissive temperatures (37 °C–39 °C), and the virus was attenuated in a mouse model of infection as compared to wild-type PR8 (PR8/WT) [35].

To evaluate if the *ts, ca* and *att* mutations of the Russian Len MDV [34] resulted in a similar phenotype in the backbone of PR8 (named henceforth PR8/Len), we introduced the amino acid substitutions into PR8’s PB2 (V478L), PB1 (K265N and V591I) and NEP (M100I) (Figure 1A) and tested them in a minigenome assay (Figure 1B,C). Green Fluorescence Protein (GFP) and Gaussia luciferase (Gluc) expression levels induced by PR8/WT polymerase were similar at the three temperatures assayed (33 °C, 37 °C and 39 °C). In the case of cells transfected with the PR8/AA polymerase complex, GFP and Gluc expression were reduced at 37 °C and, more notably, at 39 °C in comparison with the expression levels at 33 °C (~ 50% and ~ 70% reduction, respectively). Likewise, GFP and Gluc expression levels in cells transfected with the PR8/Len polymerase were highly reduced at 37 °C and almost undetectable at 39 °C as compared to the levels observed at 33 °C. These results demonstrate that the mutations of the Russian Len MDV induce a more robust temperature sensitive phenotype than the mutations of the US AA MDV in the context of the viral polymerase complex of PR8.

**Figure 2:**
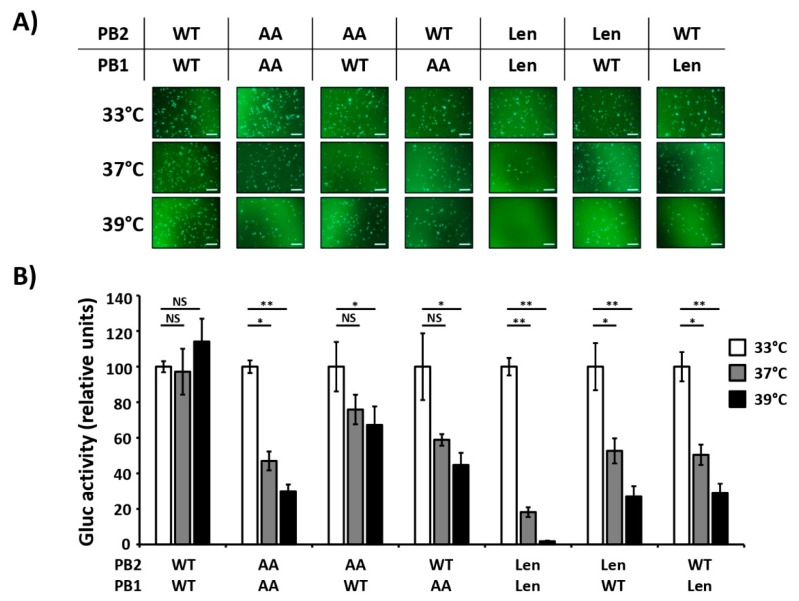
Contribution of PB2 and PB1 mutations of PR8/AA and PR8/Len in the viral polymerase activity at different temperatures. HEK293T cells (12-well plate format, 4 × 10^5^ cells/well, triplicates) were transiently co-transfected with 250 ng of the indicated combinations of ambisense pDZ expression plasmids encoding the PB2 and PB1 from PR8/WT, PR8/AA and PR8/Len together with the pDZ encoding PA and NP PR8/WT proteins; together with 500 ng of the hPol-I-GFP (**A**) and -Gluc (**B**), and 100 ng of SV40 Cluc to normalize transfection efficiencies. After transfection, cells were placed at 33 °C, 37 °C, or 39 °C, and reporter gene expression was evaluated 24 h later by GFP imaging (**A**) and Gluc expression (**B**). Gluc activity was normalized to that of Cluc. Data represent means and SDs of the triplicates. Normalized reporter expression is relative to that in the absence of pDZ NP plasmid. Data were represented as relative activity considering the activity of each polymerase complex at 33 °C as 100%. *, *P* < 0.05; **, *P* < 0.01; NS, no statistical differences (Student *t* test). Scale bars, 200 μm.

Both of the commercial/licensed lineages of LAIV (AA and Len) have mutations in the PB2 and PB1 viral segments. Thus, we next evaluated which of the viral segments has a more significant impact on the temperature sensitive phenotype in PR8. Using the same minigenome approach and different combinations of PB2 and PB1 viral segments of PR8/WT, PR8/AA or PR8/Len, GFP (Figure 2A) and Gluc (Figure 2B) expression levels were evaluated. PR8/WT polymerase activity was similar at the three tested temperatures (33 °C, 37 °C and 39 °C), while reporter gene expression by PR8/AA and PR8/Len polymerase complexes was reduced at 37 °C and, more markedly, at 39 °C, similar to our initial minigenome studies (Figure 1). When PR8/AA PB2 or PB1 were combined with PR8/WT PB1 and PB2, respectively, we observed reduced GFP and Gluc expression levels at 37 °C and 39 °C as compared to those at 33 °C (Figure 2A,B). However, the AA PB1 mutations conferred a stronger *ts* phenotype effect than the AA PB2 mutations. In the case of PR8/Len, both the PB1 and PB2 segments induced a similar decrease in GFP and Gluc expression at 37 °C and 39 °C (Figure 2A,B). Remarkably, the combinations that contain only one of the viral segments (PB2 or PB1) with the LAIV mutations (AA or Len) had an accentuated *ts* phenotype in our minigenome assays. These results demonstrate that mutations in both PB2 and PB1 segments are responsible for the *ts* phenotype of AA and Len MDV and an additive effect in the polymerase activity at high temperatures is observed when both viral proteins (PB2 and PB1) have the aforementioned mutations.

### 3.2. Recombinant PR8/Len is More Temperature Sensitive than PR8/AA

**Figure 3:**
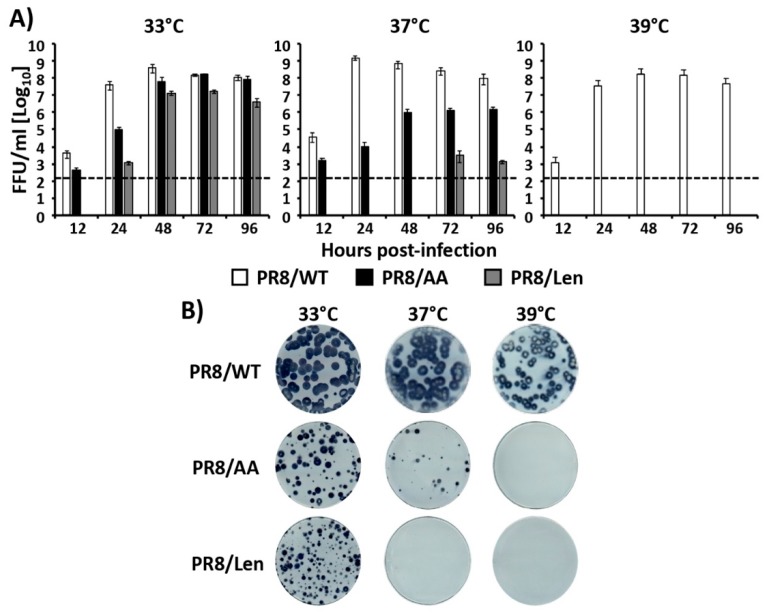
In vitro characterization of PR8/AA and PR8/Len viruses. (**A**) Multicycle growth kinetics: MDCK cells (12-well plate format, 4 × 10^5^ cells/well, triplicates) were infected at low MOI (0.001) with PR8/WT (white bars), PR8/AA (black bars) and PR8/Len (grey bars) viruses and placed at 33 °C, 37 °C, or 39 °C. Tissue culture supernatants were recovered at the indicated times post-infection (p.i.) and viral titers were determined by immunofocus assay (FFU/mL) using an anti-NP mAb (HB-65). Data represent the means and SDs of the results determined from triplicate wells. Dotted black lines indicate the limit of detection (200 FFU/mL). (**B**) Plaque assays: MDCK cells (6-well plate format, 1 × 10^6^ cells/well) were infected with PR8/WT, PR8/AA or PR8/Len viruses and incubated at 33 °C, 37 °C, or 39 °C for 3 days. Plaque phenotype was assessed by immunostaining with the HB-65 anti-NP mAb.

Based on the minigenome results using transfected cells in which Len MDV mutations induced a stronger reduction in the PR8 polymerase activity at high temperatures (37 °C and 39 °C) than those induced by the AA MDV mutations (Figure 1 and Figure 2), we next evaluated the impact of the AA and Len MDV mutations in the context of PR8 viral infections. To that end, we used reverse genetics [43] to rescue a PR8/Len virus and then evaluated and compared the viral replication kinetics (Figure 3A) and plaque phenotype (Figure 3B) of PR8/Len with those of PR8/WT and our previously described PR8/AA [35] at different temperatures (33 °C, 37 °C and 39 °C). At 33 °C, PR8/WT reached higher viral titers than PR8/AA and PR8/Len viruses at early times (12 h and 24 h) post-infection (p.i.), but at 72 h p.i. the three viruses reached similar viral titers (Figure 3A). Replication of PR8/WT at 37 °C and 39 °C was similar to that observed at 33 °C. However, as previously described [35], PR8/AA replication was reduced at 37 °C, and abolished at 39 °C. In the case of PR8/Len, replication was only detected at the latest time p.i. (72 h) at 37 °C, and virus was not detected at 39 °C. Likewise, the plaque phenotype of PR8/WT was similar at 33 °C and 37 °C and slightly reduced at 39 °C (Figure 3B), in agreement with the results of the growth kinetic studies (Figure 3A). In the case of PR8/AA, both the number and the size of the plaques were reduced (37 °C), or not detected (39 °C), when compared to 33 °C. PR8/Len could only generate plaques at 33 °C, corroborating the growth kinetics results (Figure 3A). Taken together, these results demonstrate that the mutations of the Russian Len MDV induce a more clear temperature sensitive phenotype in the backbone of PR8 than the mutations of the US AA MDV.

### 3.3. Attenuation of PR8/Len and PR8/AA in a Mouse Model of Influenza Infection

**Figure 4:**
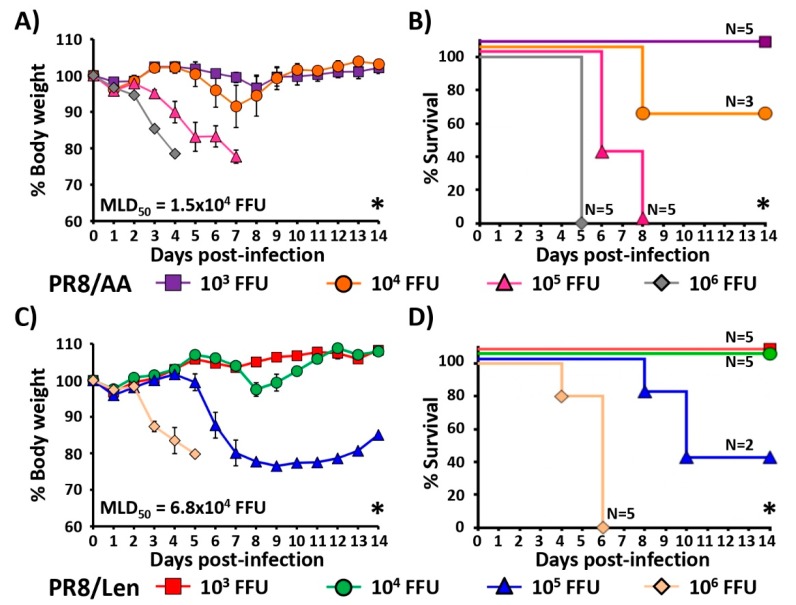
Attenuation of PR8/AA and PR8/Len viruses in vivo. Six-to-8-week-old female C57BL/6 mice (*n* = 5) were infected intranasally (i.n.) with the indicated FFU of PR8/AA (**A**,**B**) or PR8/Len (**C**,**D**) viruses and then monitored daily for 2 weeks for body weight loss (**A**,**C**) and survival (**B**,**D**). Mice that lost 25% of their initial body weight were sacrificed. Data represent the means and SDs of the results determined for individual mice (*n* = 5). The viral 50% mouse lethal dose (MLD_50_) for PR8/AA and PR8/Len were calculated based on survival data using the method of Reed and Muench [50]. (**A**,**C**) Statistical analysis was performed using one-way ANOVA test comparing % body weight loss between groups (* *P* < 0.0001). (**B**,**D**) Statistical analysis was performed using log-rank (Mantel-Cox) test (* *P* < 0.0001).

In our next set of experiments, we compared the virulence of both PR8 LAIVs in vivo (Figure 4). To that end, groups of C57BL/6 mice (*N* = 5) were infected with 10^3^, 10^4^, 10^5^ and 10^6^ fluorescent forming units (FFU) of PR8/AA (Figure 4A,B) or PR8/Len (Figure 4C,D) and monitored over 14 days p.i. for body weight loss (Figure 4A,C) and survival (Figure 4,D). As previously described [35], mice infected with 10^5^ and 10^6^ FFU of PR8/AA showed a dramatic weight loss at early times p.i. (Figure 4A), resulting in a rate of mortality of 100% at days 5 and 8 p.i., respectively (Figure 4B). Two mice (40%) infected with 10^4^ FFU of PR8/AA lost weight and died at day 8 p.i., while mice infected with 10^3^ FFU of PR8/AA virus did not show noticeable weight loss and all of them survived infection, resulting in a theoretical 50% mouse lethal dose (MLD_50_) of ~1.5 × 10^4^ FFU, as previously described [50]. In the case of PR8/Len (Figure 4C,D), all mice infected with 10^6^ FFU died at day 6p.i., 40% of mice survived infection with 10^5^ FFU and those infected with 10^4^ and 10^3^ FFU did not show body weight loss and all survived viral infection, resulting in a calculated MLD_50_ of ~6.8 × 10^4^ FFU (i.e., ~5 fold greater attenuation than PR8/AA) [50].

**Figure 5:**
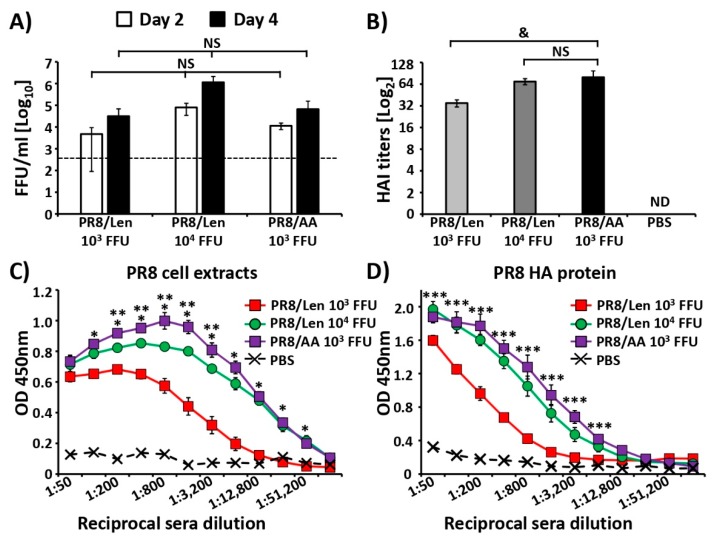
Replication of PR8/AA and PR8/Len viruses in mice and induction of humoral immune responses. (**A**) Lung viral titers: Six-to-eight-week-old female C57BL/6 mice (*n* = 6) were infected i.n. with 10^3^ and 10^4^ FFU of PR8/Len and 10^3^ FFU of PR8/AA viruses. Viral titer in lungs of infected mice was evaluated at days 2 and 4 p.i. (*n* = 3) by immunofocus assay (FFU/mL) in MDCK at 33 °C using an anti-NP mAb (HB-65). Data represent the means and SDs of the results determined from each group of mice (*n* = 3). Dotted black line represents the limit of detection of the assay (200 FFU/mL). NS, no statistical differences (Student *t* test). (**B**–**D**) Humoral immune responses: Six-to-eight-week-old female C57BL/6 mice (*n* = 5) were infected i.n. with 10^3^ and 10^4^ FFU of PR8/Len and 10^3^ FFU of PR8/AA viruses, or mock-infected (PBS). At 14 days p.i., mice were bled and sera were collected and evaluated individually by HAI assay for hemagglutination inhibiting antibodies (**B**) and by ELISA for IgG Abs against total influenza viral proteins using cell extracts of MDCK cells infected with PR8/WT (**C**) or against recombinant purified PR8 HA protein (**D**). (**B**) HAI titers: ND, not detected. &, *p* < 0.05 (PR8/Len 10^3^ FFU vs PR8/AA 10^4^ FFU) (Student *t* test); NS, no statistical differences (Student *t* test). (**C**,**D**) ELISAs: OD, optical density. Data represent the means and SDs of the results for five individual mice. *, *p* < 0.001 (PR8/Len 10^3^ FFU vs.PR8/AA 10^3^ FFU); **, *p* < 0.05 (PR8/Len 10^4^ FFU vs.PR8/AA 10^3^ FFU); ***, *p* < 0.01 (PR8/Len 10^3^ FFU vs.PR8/AA 10^3^ FFU) (Student *t* test).

We also evaluated viral replication in the lungs of mice infected with PR8/Len and PR8/AA. To that end, groups of C57BL/6 mice (*N* = 6) were infected with 10^3^ and 10^4^ FFU of PR8/Len or 10^3^ FFU of PR8/AA (doses that resulted in 100% survival, Figure 4) and viral replication was evaluated at days 2 (*N* = 3) and 4 (*N* = 3) p.i. (Figure 5A). No statistically significant differences were observed in lung viral titers in animals infected with PR8/Len or PR8/AA, although higher viral titers were detected in the lungs of mice infected with 10^4^ FFU of PR8/Len.

### 3.4. Immune Responses and Protection Efficacy of PR8/Len and PR8/AA in Mice against a Homologous Viral Challenge

We next evaluated and compared immune responses induced by PR8/Len and PR8/AA viruses (Figure 5B,D). Groups of C57BL/6 mice (*N* = 5) were immunized with the same non-lethal doses of PR8/Len (10^3^/10^4^ FFU) and PR8/AA (10^3^ FFU) (Figure 4), or mock infected with PBS. Hemagglutination inhibition (HAI) assay results revealed a higher titer of hemagglutination inhibiting antibodies in mice vaccinated with 10^3^ FFU of PR8/AA than those vaccinated with 10^3^ FFU of PR8/Len, while no statistical differences were observed between the sera of mice immunized with 10^3^ FFU of PR8/AA and 10^4^ FFU of PR8/Len (Figure 5B). Humoral immune responses were also evaluated by ELISA using cell extracts from MDCK cells infected with PR8/WT virus (Figure 5C) or purified PR8 HA protein (Figure 5D). Sera from all virus-vaccinated mice showed antibodies against total PR8 viral proteins and against the viral HA (Figure 5C,D, respectively). Interestingly, sera from mice vaccinated with 10^3^ FFU of PR8/AA showed a significantly higher titer of antibodies than sera from mice immunized with 10^3^ FFU of PR8/Len, while comparable antibody responses were detected in the sera from mice immunized with 10^3^ FFU of PR8/AA and 10^4^ FFU of PR8/Len.

**Figure 6:**
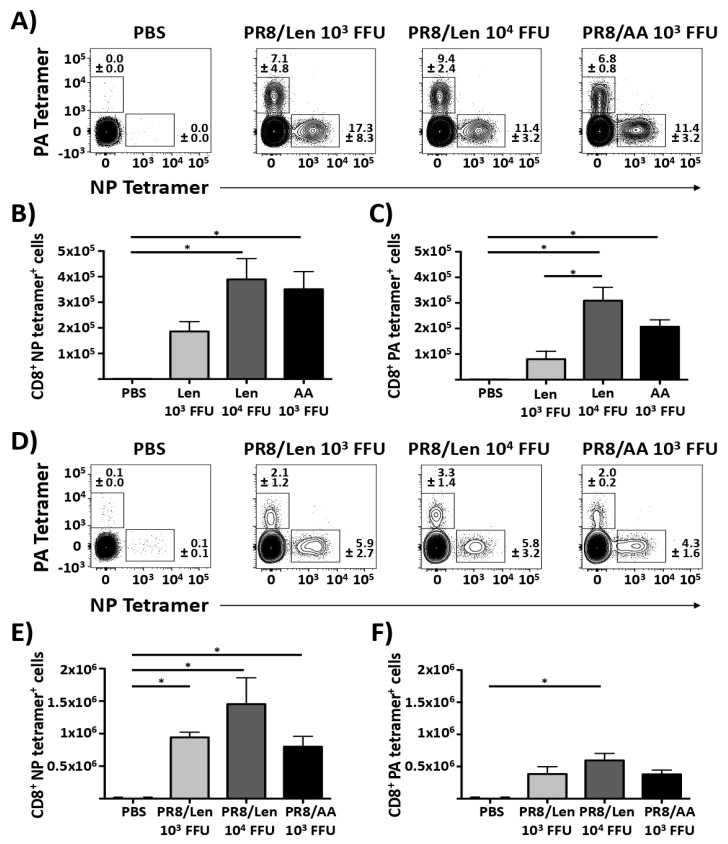
Induction of CD8 T cell responses by PR8/AA and PR8/Len viruses: Female six-to-eight-week-old C57BL/6 mice (*n* = 5) were infected (i.n.) with 10^3^ and 10^4^ FFU of PR8/Len and 10^3^ FFU of PR8/AA viruses. Mice were also mock (PBS) infected as an internal control. Ten days p.i., lungs (**A**–**C**) and spleens (**D**–**F**) were collected and resident cells were prepared for flow cytometry. Live CD8 T cells specific for NP (**B**,**E**) or PA (**C**,**F**) tetramers were counted. Data represent the means +/− SDs of the results for 5 individual mice. *, *p* < 0.001 for the indicated pair wise comparisons (Student *t* test).

To have a more complete analysis of immune responses, we also evaluated and compared the induction of CD8 T cell responses by PR8/AA and PR8/Len (Figure 6). To that end, groups of C57BL/6 mice (*N* = 5) were immunized with 10^3^ or 10^4^ FFU of PR8/Len, 10^3^ FFU PR8/AA, or mock-vaccinated (PBS). At 10 days p.i., lungs and spleens were recovered and processed to collect CD8 T cells. The results showed that vaccination with both PR8 LAIVs induced NP- and PA-specific CD8 T cells in the lungs (Figure 6A,C) and the spleen (Figure 6D,F). Although no statistical differences were observed when comparing the responses induced by PR8/Len versus PR8/AA (with the exception of the levels of PA-specific CD8 T cells in the lungs of mice vaccinated with 10^3^ and 10^4^ FFU of PR8/Len, Figure 6C), the number of CD8 T cells in the lungs and spleen tended to be greater after vaccination with 10^4^ FFU of PR8/Len and 10^3^ FFU of PR8/AA than with 10^3^ FFU PR8/Len. These results suggest that vaccination with both LAIVs elicits a robust virus-specific CD8 T cell response in the spleen and lung.

**Figure 7:**
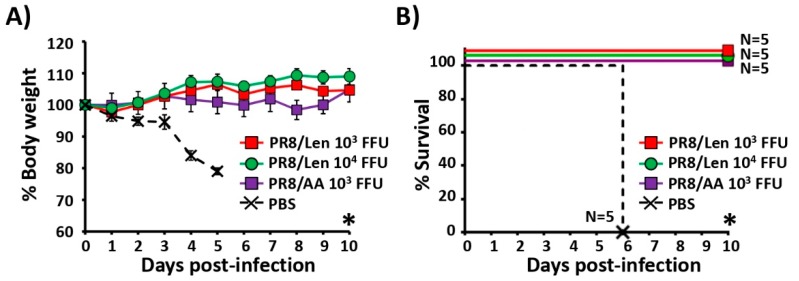
Protection efficacy of PR8/AA and PR8/Len against homologous viral challenge: Six-to-eight-week-old female C57BL/6 mice (*n* = 5) were infected i.n. with 10^3^ and 10^4^ FFU of PR8/Len and 10^3^ FFU of PR8/AA viruses, or mock vaccinated (PBS). At 15 days p.i., mice were challenged i.n. with 1,000 MLD_50_ of PR8/WT and monitored daily over 10 days for body weight loss (**A**) and survival (**B**). Mice that lost 25% of their initial body weight were sacrificed. Data represent the means and SDs of the results determined for individual mice (*n* = 5). Statistical analysis was performed using one-way ANOVA (**A**) and Dunnett’s (**B**) tests comparing the PBS group to the other groups (* *P* < 0.0001).

Having confirmed the immunogenicity of both LAIVs, we proceeded with challenge experiments, initially focusing on a homologous viral challenge (Figure 7). Groups of C57BL/6 mice (*N* = 5) were vaccinated with the same PR8 LAIVs doses used in the immunogenicity studies, or mock (PBS) vaccinated (Figure 6). At day 15 post-vaccination, mice were challenged with 1000× MLD_50_ of PR8/WT and monitored over 10 days for body weight loss (Figure 7A) and survival (Figure 7B). All mice mock vaccinated lost weight quickly and died at day 6 post-challenge. All mice vaccinated with PR8/Len (independently of the dose) and PR8/AA survived without body weight loss. These results indicate that although PR8/Len is more attenuated than PR8/AA (Figure 4), it is able to confer protection against a homologous PR8 viral challenge with the same viral dose (10^3^ FFU). Since all mice survived the challenge with PR8/WT, we cannot conclude which one of the two LAIVs (PR8/AA or PR8/Len) is more protective.

### 3.5. Immune Responses and Protection Efficacy of PR8/Len and PR8/AA Against a Heterologous Viral Challenge

**Figure 8:**
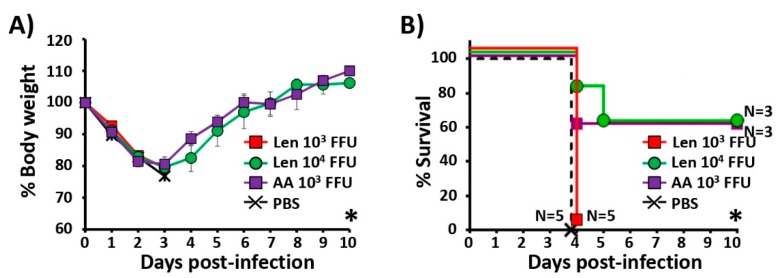
Protection efficacy induced by PR8/AA and PR8/Len against heterologous virus challenge. Six-to-eight-week-old female C57BL/6 mice (*n* = 5) were infected i.n. with 10^3^ and 10^4^ FFU of PR8/Len or 10^3^ FFU of PR8/AA viruses, or mock-infected (PBS). At 15 days p.i., mice were challenged i.n. with 50 MLD_50_ of X-31 and then monitored daily over 10 days for body weight loss (**A**) and survival (**B**). Mice that lost 25% of their initial body weight were sacrificed. Data represent the means and SDs of the results determined for individual mice (*n* = 5). Statistical analysis was performed using one-way ANOVA (**A**) and Dunnett’s (**B**) tests comparing the PBS group to the other groups (* *P* < 0.0001).

We next examined the humoral immune responses and the protection efficacy of PR8/Len and PR8/AA against a heterologous challenge with X31 (H3N2) (Figure 8). Groups of C57BL/6 mice (*N* = 5) were vaccinated with the same PR8/Len and PR8/AA viral doses used in the homologous challenge studies (Figure 7). Fourteen days post-vaccination, mice were bled and the presence of total antibodies against X31 were analyzed by ELISA. Sera from mice immunized with 10^3^ FFU of PR8/AA showed significantly higher antibody titers than sera from mice vaccinated with 10^3^ FFU of PR8/Len and, to a lower extent, than mice vaccinated with 10^4^ FFU of PR8/Len. It is worth mentioning that X31 is a PR8 virus containing the HA and NA of A/Hong Kong/68 H3N2. Contrarily, the PR8 LAIVs (both AA and Len) contain their respective H1N1 glycoproteins and, therefore, the antibodies detected in the ELISA are mainly directed against internal PR8 viral proteins, which are the same in all viruses.

Then, fifteen days post-vaccination, mice were challenged with 50× MLD_50_ of X31 H3N2 and monitored daily over 10 days for body weight loss (Figure 8A) and survival (Figure 8B). Mock-vaccinated mice and mice vaccinated with 10^3^ FFU of PR8/Len lost weight and all succumbed to viral challenge with X31 at day 4 post-challenge (Figure 8A,B). Mice vaccinated with 10^3^ FFU of PR8/AA and 10^4^ FFU of PR8/Len also lost weight (Figure 8A) but 60% of them (in both groups) recovered and survived the X31 lethal challenge (Figure 8B). These results indicate that, at the same dose, PR8/AA induces stronger immune responses and protection against a heterologous viral challenge than PR8/Len. However, induction of immune responses and protection efficacy with 10^4^ FFU of PR8/Len is equivalent to that induced with 10^3^ FFU of PR8/AA. Altogether, these results indicate that it is necessary to vaccinate mice with a higher dose (~1 log) of PR8/Len than PR8/AA to induce the same levels of protective immunity against a heterologous challenge.

## 4. Discussion

**Figure 9:**
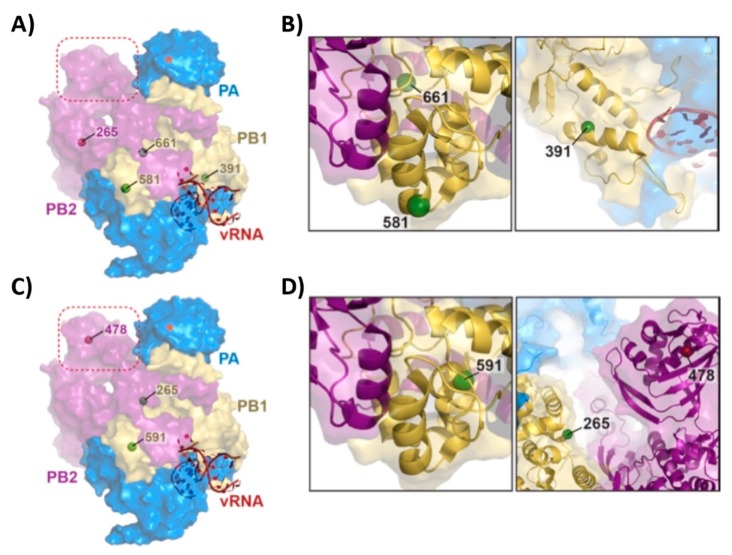
Location of the *ts, ca* and *att* mutations of AA and Len MDV in the viral polymerase complex. (**A**) PB1 (green spheres) and PB2 (purple sphere) mutations responsible for the ts, ca and att phenotype of AA are shown on the reference model of the RNA dependent RNA polymerase, RdRp, complex (PDB ID 5M3H) [58]. The RdRp complex is shown as a surface-rendering model with its subunits PA (blue), PB1 (yellow) and PB2 (purple), and bound vRNA (red). Dotted square (red) indicates the PB2 cap-binding domain. Red asterisk shows the approximate location of the endonuclease catalytic site of PA. (**B**) Close-up images of the locations of the AA PB1 mutations E581G and A661T (left) and K391E (right). (**C**) Same as in (**A**) but for Len. (**D**) Close up locations of LenV591I (PB1, left), K265N (PB1, right) and V478L (PB2, right) mutations. All images were generated and rendered in the program Pymol [59].

The mutations responsible for the *ts, ca* and *att* phenotypes of the two commercially available MDVs (US (AA) and Russian (Len)) have been previously described [22,24,30,53,54,55,56,57]. One mutation in PB2 (N265S), three substitutions in PB1 (K391E, E581G and A661T) and one amino acid change in NP (D34G) are the major contributors to the *ts, ca* and *att* phenotype of US AA MDV. Mutations located in the PB2 (V478L), PB1 (K265N and V591I) and in the NEP (M100I) are responsible for the *ts, ca* and *att* phenotype of the Russian Len MDV (Figure 9). Using a bat influenza A virus polymerase structure solved by X-ray crystallography as a reference model (PDB ID 5M3H, [58]), which is homologous to H1N1 virus polymerase complex with greater than 80% sequence similarity (PB2: 83%, PB1: 89% and PA: 83%), the location of these mutations are identified as distal from the endonuclease catalytic site of the PA subunit and appear rather random. However, upon closer inspection, we realize many of these mutations are at or near the interface between subunits, in particular, A661T mutation present in the PB1 from US AA MDV, and V591I and K265N mutations found in the PB1 from Russian Len MDV (Figure 9B,D, respectively). Therefore, we speculate that the mutations responsible for the *ts*, *ca* and *att* phenotype may relate to their effect on polymerase complex formation, possibly affecting the stability of the complex, rather than affecting the stability of each subunit itself. Of particular note, it is interesting to see the presence of a mutation within the cap-binding domain of PB2 subunit (V478L, Figure 9C), possibly affecting its cap-snatching activity to generate a 5’-capped primer for transcription initiation. This mutation is only found in the Russian Len MDV, and perhaps explains the difference seen in its attenuation when compared to that of US AA MDV. However, further experiments are necessary to elucidate the roles of the LAIV mutations and to gain a better mechanistic understanding of these attenuated LAIVs.

Since a direct comparison of the mutations responsible for the *ts, ca* and *att* phenotype of the US AA and the Russian Len MDVs in the same virus backbone had not been conducted, we introduced the mutations of the Russian Len MDV into PR8, and compared them to our previously described PR8 containing the US AA MDV mutations [35]. Using a minigenome assay, we observed that the mutations of the Russian Len MDV resulted in a greater reduction in polymerase activity at non-permissive (37ºC and 39ºC) temperatures compared to those of the US AA MDV (Figure 1). Similar results were obtained when the replicative phenotype of a recombinant PR8 containing the mutations in the PB2, PB1 and NEP of the Russian Len MDV (PR8/Len) was examined, and compared to our previously described PR8/AA [35]. In vitro results (multicycle growth kinetics and plaque phenotype) demonstrated that PR8/Len was more attenuated at high temperatures (37 °C and 39 °C) than PR8/AA (Figure 3), similar to our minigenome results (Figure 1 and Figure 2). Analogous results were obtained in vivo since PR8/Len was more attenuated than PR8/AA, with the PR8/Len MLD_50_ being ~5× greater than the MLD_50_ of PR8/AA (Figure 4).

When we compared the ability of PR8/AA and PR8/Len to induce protective immune responses against homologous (Figure 5) and heterologous (Figure 8) viral challenges, as well as CD8T cell responses (Figure 6), we found that it was necessary to immunize mice with a ~ 1 log higher dose of PR8/Len (10^4^ FFU) than PR8/AA (10^3^ FFU) to reach similar levels of humoral (Figure 5 and Figure 8) and cellular (Figure 6) responses. Notably, vaccination with 10^3^ FFU of PR8/Len induced complete protection against a homologous challenge with PR8 (Figure 7), while similar levels of protection against heterologous challenge with X-31 were observed using 10x more PR8/Len than PR8/AA (Figure 8). Although no statistically significant differences were observed in the levels of influenza-specific CD8 T cells induced with 10^3^ or 10^4^ FFU of PR8/Len, or 10^3^ of PR8/AA (Figure 6A–C), levels of positive CD8 T cells in the lungs from mice vaccinated with 10^4^ FFU PR8/Len and 10^3^ FFU PR8/AA were similar - and greater than those observed in mice infected with 10^3^ FFU of PR8/Len, correlating with the level of protection against X31 challenge (Figure 8).

Collectively, these results demonstrate that the mutations responsible for the *ts, ca* and *att* phenotypes of the US AA MDV can attenuate a broad range of other influenza viral strains, including human [35], canine [11,12,21] and equine [13] IAVs. Likewise, the mutations of the Russian Len MDV conferred robust *ts, ca* and *att* phenotypes in the genetic background of PR8. It remains to be determined whether this also applies to different influenza virus strains and/or subtypes. Nevertheless, our results have important implications for the development of new and improved LAIVs for the treatment of human and/or animal influenza infections. Specifically, our findings with PR8 suggest that it may be possible to update the MDV backbone of the currently available US or Russian H2N2 LAIVs by simply introducing the mutations of the Russian Len or US AA MDVs into the backbone of a more recent H1N1 (and maybe H3N2) seasonal circulating strain. However, it will be important to demonstrate that introduction of these *ts, ca* and *att* mutations results in the desired attenuated phenotype since we have previously shown that introduction of the US AA MDV mutations into the backbone of a recent pandemic Cal/09 did not resulted in an attenuated phenotype in vivo [37,38,39].
